# Sigmoid intussusception from submucosal lipoma in an adolescent

**DOI:** 10.1093/jscr/rjag389

**Published:** 2026-05-27

**Authors:** Syed Saad Ali Chishti, Rimsa Tahir

**Affiliations:** Department of Colorectal Surgery, Beaumont Hospital, Beaumont Road, Beaumont, Dublin 9, D09 V2N0, Ireland; Cavan General Hospital, Cavan, County Cavan, H12 A5D7, Ireland

**Keywords:** intussusception, colonic lipoma, adolescent, submucosal lipoma, bowel obstruction, sigmoid colectomy

## Abstract

Colo-colic intussusception secondary to a submucosal lipoma is exceedingly rare, particularly in adolescents, and presents significant diagnostic and management challenges. A 16-year-old male presented with a 5-day history of left lower quadrant pain, nausea, vomiting, and currant jelly stool. Abdominal ultrasound identified a left iliac fossa mass, and contrast-enhanced computed tomography confirmed a submucosal lipoma with a 10 cm colo-colic intussusception and pelvic free fluid. Conservative management via contrast enema and sigmoidoscopy failed due to mucosal oedema and ulceration, and open sigmoid colectomy was performed. Histopathology confirmed a 32 mm mature lipoma as the lead point with no evidence of malignancy. The patient recovered well and was discharged on postoperative day ten. This case highlights that colo-colic intussusception in adolescents should not be presumed idiopathic. A pathological lead point must always be excluded, and when conservative measures fail, timely surgical resection ensures definitive treatment and favourable outcomes.

## Introduction

Although intussusception is amongst the most frequent causes of bowel obstruction in childhood, its occurrence beyond early childhood is uncommon, with adult cases contributing to ˂1% of all intestinal obstructions. [1, 2] In contrast to the paediatric population, adult intussusception is strongly associated with an underlying structural cause, and a malignant aetiology accounting for 50%–60% of large bowel cases, whilst benign causes such as lipoma represent a considerably smaller proportion [3].

Colonic submucosal lipomas are benign adipose tumours with a low overall prevalence of 0.035%–4.4%, yet represent one of the most common benign tumours of the large bowel, with the colon accounting for 65%–75% of all gastrointestinal lipomas [2, 4].Whilst most remain clinically silent, a small subset precipitate acute presentations through lipoma-induced intussusception [4, 5].

Submucosal lipoma as a precipitant of colo-colic intussusception in the adolescent age group is an exceptionally uncommon clinical entity, with the published literature limited to isolated case reports. This case is presented to draw attention to the diagnostic and therapeutic challenges involved, and to advocate for a staged, conservative-first management strategy when pre-operative imaging points towards a benign aetiology [1, 5].

## Case presentation

A 16-year-old male was referred to the Acute Surgical Assessment Unit following a 5-day history of left lower quadrant pain, accompanied by nausea, vomiting, and anorexia, with currant jelly stool developing on the day of admission. Abdominal examination revealed localized tenderness over the left iliac fossa without peritonism. Admission bloods were unremarkable and are detailed in Table 1.

**Table 1:** Summary of admission laboratory investigations.

**Investigation**	**Result**	**Normal Range**
Haemoglobin	14.8 g/dL	13.0–17.0 g/dL
C-reactive protein	<5 mg/L	<10 mg/L
White blood cell count	12.5 × 10^9^/L	4.0–11.0 × 10^9^/L
Lactate	0.9 mmol/L	<2.0 mmol/L

**Figure 1:**
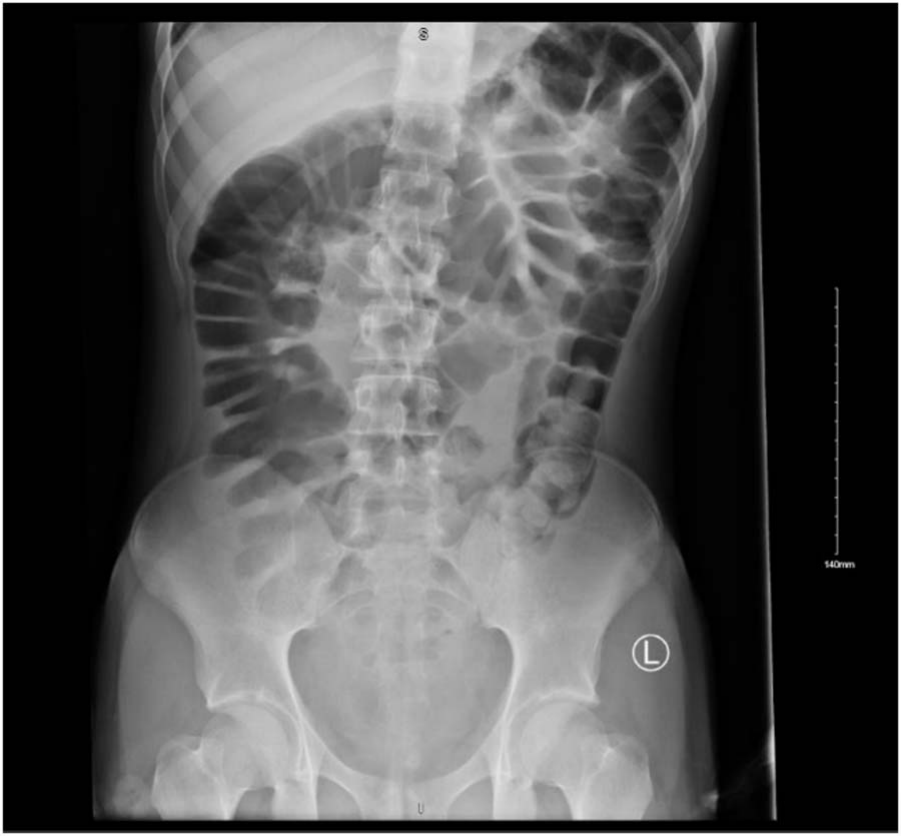
Plain abdominal radiograph demonstrating colonic distension and faecal loading of the sigmoid colon.

**Figure 2:**
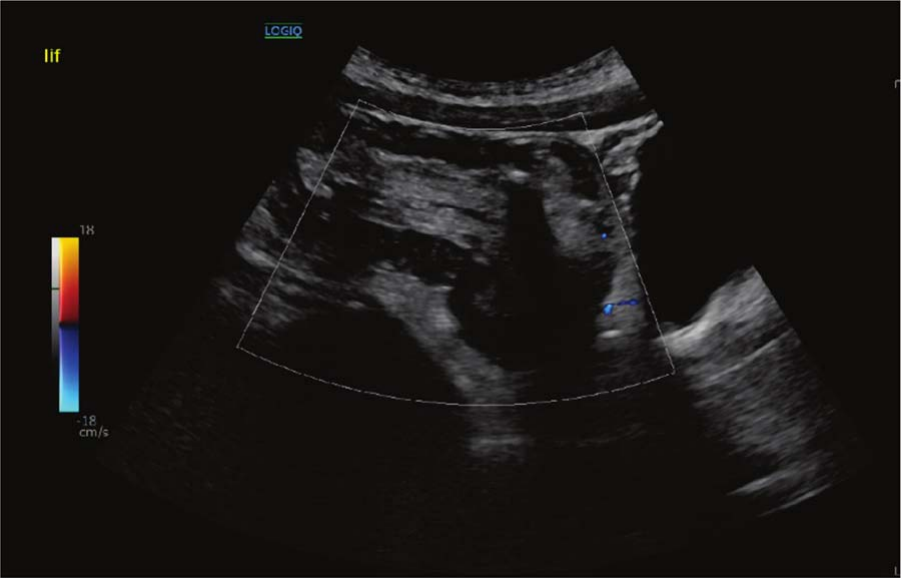
Abdominal ultrasound demonstrating a left iliac fossa mass suggestive of colo-colic intussusception.

**Figure 3:**
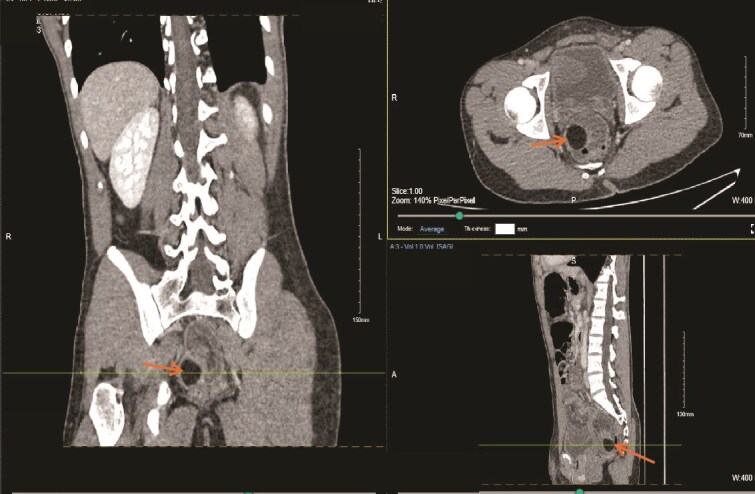
Contrast-enhanced CT scan of the abdomen demonstrating a 24 mm fat-density submucosal lesion consistent with a lipoma acting as the lead point for a 10 cm colo-colic intussusception, with associated pelvic free fluid.

On initial assessment, abdominal X-ray (Fig. 1) showed a distended colon and faecal loading of the sigmoid colon. Following this, an abdominal ultrasound (Fig. 2) was performed, which demonstrated a left iliac fossa mass suggestive of colo-colic intussusception. A subsequent contrast-enhanced computed tomography (CT) scan (Fig. 3) demonstrated a fat-density submucosal lesion measuring 24 mm, consistent with a lipoma, with a 10 cm colo-colic intussusception and free fluid in the pelvic cavity. The larger measurement of 32 mm on histopathology reflects the known difference between in-vivo compressed imaging dimensions and ex-vivo specimen size.

**Figure 4:**
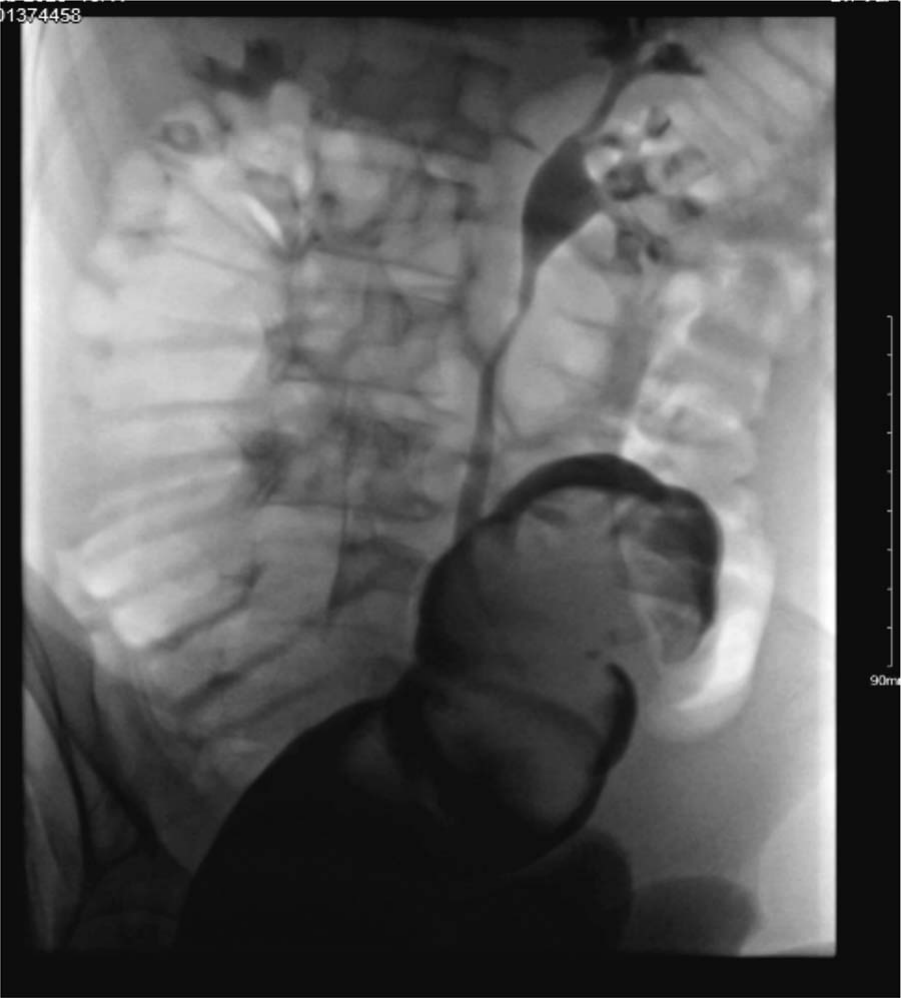
Urograffin enema demonstrating unsuccessful attempt at non-operative decompression of the intussusception.

A decision was made to decompress the intussusception using a urograffin enema (Fig. 4); however, this was unsuccessful. Another attempt was made via sigmoidoscopy in the operating theatre (OT) as a final attempt at conservative management. However, due to extensive mucosal ulceration and mucosal oedema, the procedure was terminated, and an open sigmoid colectomy was performed. Histopathological examination of the resected sigmoid colon, measuring 32 cm in length and 10 cm in diameter, confirmed intussusception with a mature lipoma, measuring 32 mm in maximum dimension, as the lead point. No evidence of malignancy or dysplasia was identified. The postoperative course was uneventful, with no complications recorded. The patient tolerated diet, mobilized well, and was discharged home in good condition on the tenth postoperative day.

## Discussion

In the paediatric age group, ileo-colic intussusception without an identifiable cause predominates. Beyond infancy, however, a structural lead point becomes increasingly probable, with colonic polyps, Meckel’s diverticulum, and Peutz-Jeghers syndrome amongst the recognized precipitants [6]. Colo-colic intussusception remains distinctly uncommon in this age group, and when encountered in an adolescent, active exclusion of an underlying lesion is mandatory rather than optional. To date, published documentation of such specific condition remains confined to isolated case reports, affirming its singular clinical rarity [3].

Choosing the appropriate imaging investigation is central to management. Sonographic assessment is favoured in younger patients to avoid ionizing radiation exposure. However, whilst it can identify intussusception, definitive characterization of the underlying lead point is often not achievable with sonography alone [1, 2, 7]. In our patient, cross-sectional CT was obtained in light of diagnostic uncertainty and identified a 24 mm fat-density submucosal mass serving as the lead point for a 10 cm colo-colic intussusception, with pelvic free fluid also noted, providing the diagnostic clarity required to proceed. Whilst endoscopic assessment carries both diagnostic and potential therapeutic value, the degree of mucosal breakdown encountered after 5 days of obstruction precluded safe instrumentation in this case [8].

Where a pathological lead point is identified or conservative reduction is unsuccessful, operative intervention is widely regarded as the definitive treatment, and resection is generally advised once a lesion exceeds 2 cm given the risk of ongoing complications [3, 4, 8]. In the adult population, where the majority of large bowel intussusceptions carry a neoplastic cause, surgery is pursued as the first-line strategy without prior conservative attempts [5]. Our patient’s young age and the unambiguous fat-density CT appearance of the lead point together justified an initial trial of non-operative management using water-soluble contrast enema and sigmoidoscopy before committing to resection. When both measures failed in the setting of established mucosal injury, open sigmoid colectomy was carried out without further delay, and histopathological analysis confirmed a mature benign lipoma with no dysplastic or malignant change. This sequence reinforces the principle that non-operative options may appropriately precede surgery in younger patients with imaging-confirmed benign lead points, provided that failure triggers prompt escalation.

## Conclusion

Colo-colic intussusception in adolescents is rare and should not be presumed idiopathic. A pathological lead point must always be excluded. CT imaging is essential for characterizing the lesion and guiding management. Although conservative measures may be attempted in younger patients, failure to achieve reduction should prompt timely surgical intervention. This case of sigmoid colo-colic intussusception from a submucosal lipoma in a 16-year-old reinforces the need for early recognition, CT-guided diagnosis, and a low threshold for surgical escalation when conservative management fails.
